# Current Pharmacogenetic Perspective on Stevens-Johnson Syndrome and Toxic Epidermal Necrolysis

**DOI:** 10.3389/fphar.2021.588063

**Published:** 2021-04-26

**Authors:** Lin Cheng

**Affiliations:** ^1^ State Key Laboratory of Ophthalmology, Zhongshan Ophthalmic Center, Sun Yat-sen University, Guangzhou, China; ^2^ School of Ophthalmology and Optometry, Eye Hospital, Wenzhou Medical University, Wenzhou, China

**Keywords:** HLA, pathogenesis, pharmacogenetics, Stevens-Johnson syndrome, toxic epidermal necrolysis

## Abstract

Adverse drug reactions are a public health issue that draws widespread attention, especially for Stevens-Johnson syndrome (SJS) and toxic epidermal necrolysis (TEN) which have high mortality and lack of efficacious treatment. Though T-cell-mediated HLA-interacted immune response has been extensively studied, our understanding of the mechanism is far from satisfactory. This review summarizes infection (virus, bacterial, and mycoplasma infection), an environmental risk factor, as a trigger for SJS/TEN. The mutations or polymorphisms of drug metabolic enzymes, transporters, receptors, the immune system genes, and T-cell-mediated apoptosis signaling pathways that contribute to SJS/TEN are discussed and summarized. Epigenetics, metabolites, and mobilization of regulatory T cells and tolerogenic myeloid precursors are emerged directions to study SJS/TEN. *Ex vivo* lymphocyte transformation test has been exploited to aid in identifying the causative drugs. Critical questions on the pathogenesis of SJS/TEN underlying gene polymorphisms and T cell cytotoxicity remain: why some of the patients carrying the risky genes tolerate the drug and do not develop SJS/TEN? What makes the skin and mucous membrane so special to be targeted? Do they relate to skin/mucous expression of transporters? What is the common machinery underlying different HLA-B alleles associated with SJS/TEN and common metabolites?

## Introduction

**FIGURE 1:**
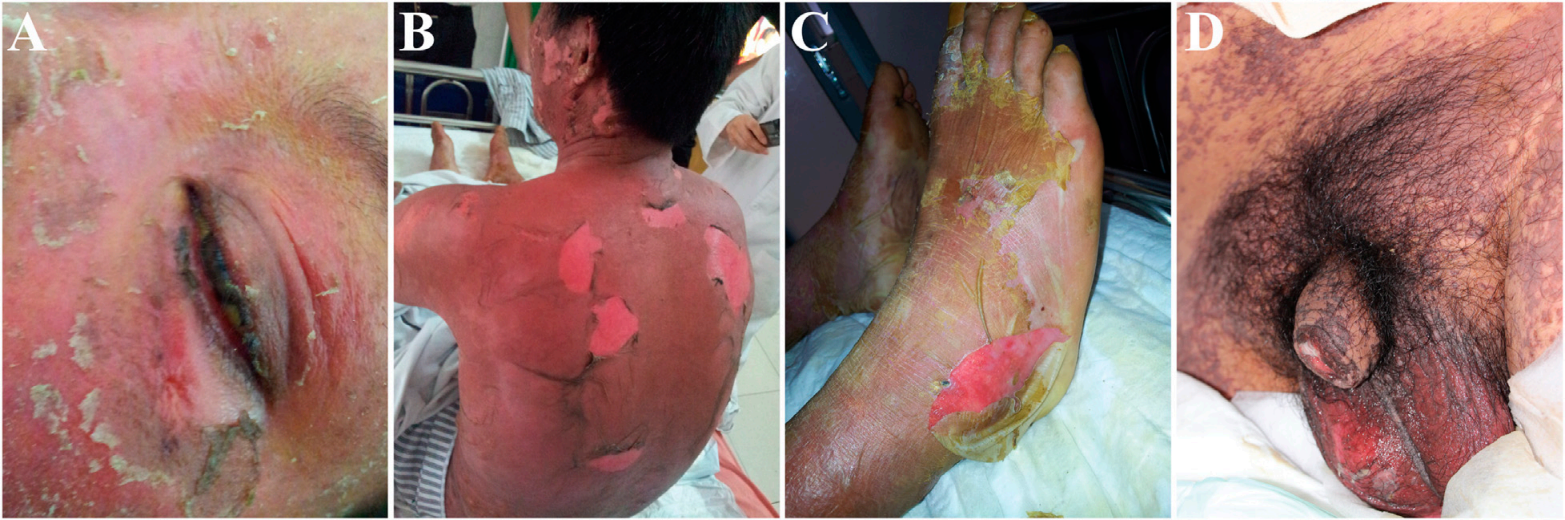
Representative images showing dermatological manifestations of patients with Stevens–Johnson syndrome (SJS) and toxic epidermal necrolysis (TEN). The patients participated in a clinical study approved by the Ethics Committee of the Institute of Clinical Pharmacology, Central South University (CTXY-110011–2). The informed consent forms were signed for the publication of the photos. **(A)** Skin erosion on the face and mucous damage in the eye; **(B)** extensive skin sloughing in the trunk; **(C)** skin peel in the feet; **(D)** painful mucous membrane damage in the genital.

Stevens–Johnson syndrome (SJS) and toxic epidermal necrolysis (TEN) are two forms of the same severe adverse drug reactions and are characterized by epidermal necrolysis. The disease has the unique expression of blisters on the skin and the affection of mucous membranes in the mouth, nose, eyes, and genitals (see Figure 1). SJS is characterized by a large area of skin and mucosal epithelial cell shedding and typical performance on the oropharynx, eyes, urogenitals, and anal mucosa. SJS has less than 10% of body surface area involvement. TEN is a more severe type than SJS and is defined as greater than 30% total body surface area skin loss. The body surface area of 10–30% is defined as SJS/TEN overlap (Duong et al., 2017). The mortality rate for SJS and TEN are as high as 1–5% and 25–30% (Harr and French, 2010; Marxer et al., 2020), respectively, and there will be a long-term multiorgan damage after acute stage. Around half patients will have both physical and psychological sequelae, including cutaneous (19.3%), oropharyngeal (4%), ocular (20.6%), urological (2.0%), gastrointestinal (0.67%), genital system (5.3%) (Wang et al., 2020b), psychological distress (71%), and impact on quality of life (Dodiuk-Gad et al., 2016).

SJS/TEN is an immune-mediated hypersensitivity and at least 200 drugs have been reported to be related to disease onset. The most common ones are nonsteroidal anti-inflammatory drugs [NSAIDs (Kowalski et al., 2013; Chong and Chao, 2017)], sulfa-derived medications [e.g., zonisamide (Vivar et al., 2018), sulphapyridine (Xiong et al., 2018), and sulfamethoxazole (Kongpan et al., 2015; Sukasem et al., 2020a; Wang et al., 2020a)], lactam antibiotics, anticonvulsants (e.g., carbamazepine, phenobarbital, and phenytoin), antiretroviral medicine, contrast agents, antigout drug allopurinol, etc. (Yang et al., 2019; Zhao et al., 2019). Some rare cases are caused by less commonly seen drugs like albuterol (Maggini et al., 2015; Shariff et al., 2017), taurine-containing energy drink (Begolli Gerqari et al., 2016), radiotherapy for cancers (Rouyer et al., 2018; Esaa et al., 2020), fertility treatment (Hashimoto et al., 2019), vaccines (Oda et al., 2017; Chahal et al., 2018; Flora et al., 2018; Su et al., 2020), commercial cannabinoid oil (Yin et al., 2020), herbs (Bonhomme et al., 2017; Lim et al., 2018), teriflunomide (treating multiple sclerosis) (Gerschenfeld et al., 2015), methotrimeprazine (Moubayed et al., 2017), diuretic drug metolazone (Kumar et al., 2016), etoricoxib (Roy et al., 2018), *Dalbergia cochinchinensis* (a tree) (Yang et al., 2015), etc. Anticancer drugs like protein kinase inhibitors ribociclib (Lopez-Gomez et al., 2019), palbociclib (Karagounis et al., 2018), afatinib (Doesch et al., 2016), and vemurafenib (Arenbergerova et al., 2017), immune checkpoint inhibitors (ICIs) (including cytotoxic T lymphocyte associated antigen-4 [CTLA-4: monoclonal antibody ipilimumab (Dika et al., 2017)], programmed cell death protein [PD-1: monoclonal antibody nivolumab (Nayar et al., 2016; Salati et al., 2018; Dasanu, 2019), pembrolizumab (Lomax et al., 2019)], programmed cell death ligand 1 [PD-L1: monoclonal antibody atezolizumab (Chirasuthat and Chayavichitsilp, 2018)], and CC chemokine receptor 4 targeting antibody mogamulizumab (Tanba et al., 2016) are also reported to cause SJS/TEN. Cancer patients are at a higher risk to develop SJS/TEN not only due to consequence of the nature of neoplastic diseases, but also exposure to a line of anticancer drugs and disruption of immune system. These anticancer drugs may trigger an abnormal cytotoxic T lymphocyte response, predisposing them to SJS/TEN.

The pathogenesis of SJS/TEN is generally considered to be cytotoxic T-cell-mediated HLA-dependent drug hypersensitivity. Drug metabolites bind with HLA protein in the body, leading to cell toxicity by killing autogenous cells. Though numerous studies show the disease is related to different genes, the underlying mechanism is not fully elucidated. It is usually described as the complex interactions among multiple gene variants and environmental factors, as well as possibly the biochemical and immunological reactions with drug metabolites. To overview the current understanding of SJS and TEN, this review summarizes the past understanding and recent advances on pharmacogenetics of drug-induced SJS and TEN, discusses unresolved questions, and foresees potential targets for prevention and treatment of the disease.

## Infection and SJS/TEN

Notwithstanding the barriers to identify environmental risk factors, infections have been identified to be associated with SJS/TNE in multiple studies. Varying levels of support suggest involvement of virus, bacteria, and other infections as a trigger for the development of SJS/TEN, though at less frequency levels. The fact that some people vaccinated with meningococcal B vaccine, yellow fever vaccination, influenza vaccination, or virus/bacterial infection had developed SJS or TEN (Oda et al., 2017; Chahal et al., 2018; Flora et al., 2018; Su et al., 2020) indicates that the activated or inactivated microorganisms (vaccine) can cause SJS/TEN. This highlights infection as the new etiology to SJS/TEN. Some may argue that the additives in the vaccine are the one that cause SJS/TEN (Oda et al., 2017). Indeed, this may be true. However, the majority of patients injected with the vaccine with the additives did not develop drug hypersensitivity, indicating the additive is safe in some extent. In addition, the underlying infection coinciding with drug reaction might rationalize the involvement of infection. The concept is that the infection will trigger the nondrug specific immune activation and influence drug metabolism or presentation, making patients more prone to drug-initiated reaction. First, the pathophysiological status of infection itself might be involved in the SJS/TEN development, as demonstrated in studies that found a significantly positive association between infection and SJS/TEN seriousness (Imatoh et al., 2017; Okamoto-Uchida et al., 2017). Second, T cell cross-reactivity against pathogen epitope and drug would affect the total balance of immunity or trigger the memory CD8^+^ T cells. This will cause an undesired immune response (Okamoto-Uchida et al., 2017), resulting in SJS/TEN. Third, the conserved etiologic agents could cooperate with drugs to form virus-drug-host complex and then trigger SJS/TEN (Shiohara and Kano, 2007; Ban et al., 2016). Fourth, the heterologous immunity model has been proposed to explain some infection triggered SJS/TEN (White et al., 2015). In this model, patients carrying a specific HLA risk allele infected by pathogen (e.g., human herpesvirus) generate pathogen-specific memory T cells. These memory T cells were inhabited at specific anatomic sites. Upon drug intake, these preexisting memory T cells become activated through cross-reaction with drugs to trigger SJST/TEN.

Together, the viral, bacterial, and prokaryotes infections play a nonnegligible role in the etiopathogenesis of SJS/TEN.

### Virus Infection

The viral infection such as human immunodeficiency virus (HIV), herpes simplex virus (HSV), Epstein-Barr virus (EBV), influenza virus, cytomegalovirus (CMV), and coxsackievirus are reported to be associated with SJS/TEN. Studies reporting the occurrence of drug-induced SJS/TEN is approximately 1,000 times (Mittmann et al., 2012) higher among HIV-infected individuals than noninfected. Coxsackievirus A6 (CA-6) which belongs to enterovirus could induce blistering skin reactions mimicking erythema multiforme major or SJS (Chung et al., 2013). Herpesvirus 7 infection was identified to cause TEN by both PCR and lymphocyte transformation test (Shen et al., 2020). It was reported that in a SJS patient without any imputable medication but showing atypical activated T lymphocytes, EBV was the one contributing to the disease (Brunet-Possenti et al., 2013). A minority of SJS cases were related to HSV infection (Lerch et al., 2018). Case studies reported that children or teenagers with influenza B infection rapidly developed SJS (Tamez et al., 2018; Goyal and Hook, 2019). These observations lead to the question whether virus infection predispose patients more to immunopathogenesis of SJS/TEN.

How could viruses trigger SJS/TEN? Studies reported that the immune response after EB virus and human herpesvirus (HHV) 6, HHV7 infection helped clear up the virus, which could make the body at a relatively high sensitivity state that makes it possible for subsequent drug therapy prone to super-react (Yoshikawa et al., 2006; Hashizume et al., 2013). Another direct evidence is the production of cytokines/chemokines such as interferon γ (IFN-γ). Subsequently high levels of IFN-γ after viral stimulation disrupted the immune balance, leading to upregulation of the major histocompatibility complex body (MHC) II molecules in antigen-presenting cells which promoted the presentation of drug hapten (Carr et al., 1994). There is another view that reactivation of the original latent virus in the body is the main reason for the development of drug eruption. The theory thought there is a delayed recurrent drug eruption after the virus is activated and the full antigen is formed (Torres et al., 2009). Unfortunately, I did not find any large scale clinical reports showing direct evidence that SJS/TEN is induced by viral infection. In contradiction, a recent study did a systematic review and found there was no association between SJS and vaccination. The reported SJS cases following vaccines, such as influenza vaccine, smallpox, anthrax and tetanus vaccine, MMR vaccine, varicella vaccine, DTaP-IPV vaccine or rabies vaccine, were not statistically significantly associated with SJS (Grazina et al., 2020). If viruses can interact with the immune system and trigger severe cutaneous adverse reactions (SCARs), the deactivated virus vaccines might have the same function, at some extent, to interact with the immune system and trigger SCARs. Whether virus initiates SJS/TEN needs further investigation.

Collectively, the interaction between viral infection and SJS/TEN is not fully understood and the underlying mechanism needs further investigation. However, on basis of available evidence, it is undeniable that virus infection plays a role in SJS/TEN formation and this may be an interest of future research.

### Bacterial Infection

Given the association between the onset of SJS/TEN and bacterial infection, it is possible that bacterial infection plays a role in the occurrence of SJS/TEN. Limited cases reported that SJS/TEN are induced by bacterial infection, such as *streptococcus* and *meningococcus* infection (Czajkowski et al., 2007; Leaute-Labreze et al., 2000). And it seems viral infection is more common than bacterial infection in SJS/TEN. Patients who had septicemia or any bacterial infection are more likely [odds ratio (OR) = 4.1, 2.56, respectively] to have SJS/TEN (Hsu et al., 2016). The explanation is the toxins produced by bacteria predispose some individuals to SJS/TEN. While the mechanism is not well investigated, new evidence is expected to be disclosed.

### Prokaryotic Microbe Infection

Mycoplasma infection is the most reported prokaryotic microbe infection associated with SJS/TEN, especially in children (Meyer Sauteur et al., 2012; Tomaino et al., 2012; Amode et al., 2018; Liu and Shen, 2019; Sah et al., 2019). Mycoplasmas are ubiquitous and the smallest, free-living microorganisms. Mycoplasma pneumonia-induced SJS/TEN are frequently reported in children and adolescents.

## Genetic Variants and SJS/TEN

### Drug Metabolic Enzymes and SJS/TEN

Most common causes of SJS/TEN are drugs. Genetic variation in drug metabolic enzymes directly affects drug metabolism and metabolic pathways, and changes drug serum concentration. Generating or accumulating metabolites that are more active than the parent drugs or defect in detoxication/clearance of the original drugs will lead to SJS/TEN. Most drugs pass through the liver, where they are metabolized by phase I enzymes (cytochrome P450 (CYP), oxidoreductase, etc.) and phase II enzymes (epoxide hydrolase, glutathione s-transferase (GST), N-acetyltransferase (NAT), etc.).

CYP enzymes oxidize most drugs into chemically reactive metabolites that need to be actively detoxified and are responsible for the clearance of various compounds. For instance, nevirapine-induced SJS/TEN was found to be associated with CYP2B6 T983C. The carriers of allelic variants of CYP2B6 T983C were in 4.2-fold higher risk to develop SJS/TEN due to low clearance of nevirapine (Ciccacci et al., 2013). Studies showed the variation in drug clearance enzyme CYP2C9*3 contributed to phenytoin-related SCARs with reduced drug clearance rate (Chung et al., 2014; Tassaneeyakul et al., 2016; Wu et al., 2018; Su et al., 2019; Sukasem et al., 2020b; Fohner et al., 2020), independently of HLA-B*15:02 risk allele. Glutathione-S-transferases (GST), which is a family of metabolic enzyme, plays a crucial role in the drug detoxification and activates some chemicals in a few cases. A study in Mozambique showed the null phenotype for glutathione transferase GSTM1 (glutathione S-transferase mu 1) conferred a higher risk of SJS/TEN in HIV-infected patients treated with nevirapine (OR = 2.94), leading to a poor metabolization of nevirapine and more predisposing of the toxic effects of 12-sulfoxyl-nevirapine (Ciccacci et al., 2017). N-acetyltransferases (NATs) are polymorphic drug-metabolizing enzymes. The isoforms of NATs in human are NAT1 and NAT2. NAT2, as one of the two-phase detoxification enzymes when xenobiotics metabolize in the body, catalyzes acetyl to the functional groups of aromatic primary amine or hydrazine from acetyl coenzyme A and generates acetamide or acetohydrazide. It plays an important role in the acetylation of isoniazid, sulfamethazine, caffeine, and certain carcinogens precursors or the like. The NAT2 slow acetylator genotype is one of the predisposing factors for the development of severe cutaneous adverse reactions to drugs that require N-acetylation in these patients (Dietrich et al., 1995). Sulphonamide-induced SJS/TEN has been reported to be linked with slow acetylator due to decreased detoxication of NAT2 (Wolkenstein et al., 1995).

Some drugs are influenced by multiple metabolic enzymes. For example, carbamazepine metabolizes through microsomal epoxide hydrolase (EPHX1), CYP3A4, and uridine 5′diphosphate-glucuronosyltransferases (UGT2B7) in the body (Hung et al., 2012; He et al., 2014; Chbili et al., 2016). Firstly, carbamazepine-epoxide is produced when carbamazepine is metabolized by CYP3A4 in the liver. Then it is inverted into inactive carbamazepine diol through the microsomal epoxide hydrolase which is encoded by EPHX1 (Hung et al., 2012). Alternatively, it could be inactivated into glucuronic acid by UGT2B7 (Hung et al., 2012). Lastly, the metabolites would be eliminated from the body in urine.

In addition, renal function impairment (RFI) is strongly associated with SJS/TEN (Hung et al., 2009). Drugs such as allopurinol, water soluble antibiotics, are metabolized in the kidney, and RFI has been shown to reduce drug metabolism in the diseased kidney compared with the normal kidney (Hung et al., 2005; Stamp et al., 2016).

To conclude, the plasma concentration of drug is largely influenced by the drug metabolism enzymes. The mutation of these enzymes is responsible for the development of idiosyncratic adverse reactions like SJS/TEN. Knowledge of which metabolizing enzyme is the most important for the induction of SJS/TEN is warranted by further investigation.

### Transporters and SJS/TEN

Drug transporters refer to the proteins which have the ability to bind drugs specifically and mediate the process of pumping the drugs in or out of the cell. Therefore, the gene polymorphisms of drug transporters will affect the absorption, distribution, metabolism, and excretion of the drug. P-glycoprotein (P-gp), which is also known as multidrug resistance protein 1 (MDR1), ATP-binding cassette subfamily B member 1 (ABCB1) or cluster of differentiation 243 (CD243), is the most scrutinized plasma membrane transporter in SJS/TEN. The well-known mutation of 3435C > T in MDR1 exon 26 can lead to low expression of P-gp and drug-resistance in epilepsy, though the drug was not specified (Siddiqui et al., 2003). ABCB1 rs10276036TT genotype was associated with nevirapine-induced skin hypersensitivity (OR = 4.01) (Dhoro et al., 2013). The ATP-binding cassette (ABC) transporters play important roles in absorption and disposition of drugs in the cells. Takenaka et al. first found, except ABCG family members, ABCA, ABCB, ABCC, ABCD, ABCE, and ABCF family members were highly or moderately expressed in the skin. And there were significant interindividual variability in the expression levels of ABC transporters in the human skin, which might be associated with drug-induced skin diseases (Takenaka et al., 2013). Whether the high expression of those transporters could lead to high amount of drug in the skin via certain transporters is unknown. There is no study reporting drugs may accumulate in the skin, mucous, and recruit the cytotoxic T cells to attack. Thus, it is necessary to investigate the relationship between the onset of SJS/TEN and different expression of high-risk transporters in the skin.

A new bioinformatics approach to reanalyze the genome wide association studies (GWAS) data identified the ABC transporter and proteasome mutation may contribute to SJS/TEN (Nicoletti et al., 2015). They also found the proteasome pathway was enriched not only in genetic variants but also in genes which were differentially expressed in blister cells from skin lesions of SJS/TEN versus peripheral blood mononuclear cells (PBMC) as assessed in a previous study (Chung et al., 2008; Nicoletti et al., 2015). That proteasome complex got involved in SJS/TEN may be due to proteasome-mediated protein degradation in generating the repertoire MHC-I presented peptides. In addition, proteasome complex participates in proliferation and activation of T lymphocytes and neutrophils, which will induce the T-cell-dependent skin reaction.

### Receptors and SJS/TEN

SJS/TEN are T-cell-mediated hypersensitivities. The T-cell receptor (TCR) repertoire is characterized in some investigations to ask if a type of TCR expanded clonally is drug specific. In carbamazepine-induced SJS/TEN, it was shown that the one who has the common TCR clonotype and carbamazepine/peptide–HLA-B*15:02 complex will trigger SJS/TEN. This might explain some HLA-B*15:02 carriers are tolerant to carbamazepine since they do not have that specific TCR clonotype. It also explains why carbamazepine-specific TCR clonotypes showed cross-reactivity to oxcarbazepine, lamotrigine, and phenytoin. Studies identified preferential TCR usage, clonal expansion, and similar CDR3 (third complementarity-determining region) clonotypes in the blister cells and PBMC of patients with carbamazepine-SCARs (Chung et al., 2015; Pan et al., 2019). They conducted an adoptive transfer of this specific αβTCR-T cells to the HLA-B*15:02 transgenic mice, and these mice which received carbamazepine developed a phenotype mimicking SCARs while those mice received vehicle control drug did not show SCARs phenotypes. The HLA-B*15:02 transgenic mice which received carbamazepine but no adoptive transfer of the αβTCR-T lymphocytes also showed no phenotype of SCARs. In addition, investigators found the TCR repertoire of allopurinol-induced SCARs seems more complex and different from that of carbamazepine–SJS/TEN (Chung et al., 2015). Likewise, Xiong et al. found SJS/TEN patients had less TCR repertoire diversity and predominant shared usage of TRBV/TRBJ clonotypes. The TCR repertoire diversity of these patients showed certain association with the clinical severity of disease (Xiong et al., 2019). Moreover, it was found the TCR CDR3 shared a similar motif in metronidazole-induced SCARs patients (Yang et al., 2020).

Other receptors involved in SJS/TEN are most focused on immune system. Receptor-interacting protein kinase 3 (RIP3) is a component of TNF receptor-1 signaling complex and is an essential part of cellular machinery for necrosis. RIP3 was highly expressed in TEN patient lesions, suggesting that RIP3 is an essential actor in the programmed death and necrosis of keratinocytes (Kim et al., 2015). The serum level of RIP3 in SJS/TEN patients was associated with the amount of necrotic changes in the epidermis and could be used to predict the disease severity of SJS/TEN (Hasegawa et al., 2020). A recent report identified a network of Toll-like receptor 3 (TLR3), prostaglandin-E receptor 3 (PTGER3), and IKAROS family zinc finger 1 (IKZF1) gene polymorphisms was significantly associated with cold medicine-SJS/TEN with severe ocular surface complications (SOC), and these genes may regulate mucocutaneous inflammation. The authors postulated that they may help to predict the development of SJS/TEN with mucocutaneous inflammation including that of the ocular surface (Ueta et al., 2007; Ueta et al., 2010; Ueta et al., 2011; Ueta, 2018). They consider it as complementary association factors for cold medicine-SJS/TEN with SOC independent of HLA-A*02:06. A group from Japan found the keratinocytes exposed to PBMC supernatants from SJS/TEN were dead, while those exposed to PBMC supernatant of ordinary drug skin reactions (ODSRs) were not. Mass spectrometric analysis identified annexin A1 and its receptor formyl peptide receptor 1 (FPR1) as a key mediator of keratinocyte death and blocking annexin A1 with antibody attenuated the disease, suggesting that necroptosis pathway mediated by annexin 1 contributed to SJS/TEN (Saito et al., 2014). Further studies regarding specific receptors or receptor polymorphisms related to SJS/TEN have yet to be found.

### The Immune System and SJS/TEN

While the mechanism of SJS/TEN is not fully addressed, accumulating studies strongly support the disease is an immune-mediated hypersensitivity induced by drug presentation by human leucocyte antigen (HLA). The drug-induced SJS/TEN is strongly linked to certain HLA alleles with overwhelming activation of cytotoxic T lymphocytes. A specific MHC, which the drug or drug metabolite binds directly to, is identified to be associated with the disease.

The MHC, also named as HLA in human, is a group of related proteins encoded by MHC genes which are located on the short arm of human sixth chromosome 6p21.3 region. The HLA DNA fragment has a length of 3.6 mega base pairs (Mb), containing approximately 200 genes (Horton et al., 2004). As the most complex human genetic system currently, HLA is mainly involved in immune response. The association of HLA and drug-induced SJS/TEN has significant ethnic disparities across drugs used. Countless studies reported different HLAs have been strongly associated with SJS/TEN (shown in Table 1).

**TABLE 1:** Selected drug-induced SJS/TEN and their association with HLA.

Drug category	Drug	Associated HLA locus	Odds ratio (OR)	Ethnicity	References
Antiseizure	Carbamazepine	HLA-B*15:02	2504	Taiwan Han Chinese	Chung et al. (2004)
			1357	Taiwan Han Chinese	Hung et al. (2006)
			25.5	Thai	Locharernkul et al. (2008)
			54.76	Thai	Tassaneeyakul et al. (2010)
			9.54	Thai	Sukasem et al. (2018a)
			71.4	Indian	Mehta et al. (2009)
			16.15	Malaysian	Chang et al. (2011)
			184	Central Chinese	Wu et al. (2010)
			114.826	Southern Han Chinese	Wang et al. (2011)
			152	Central and northern Han Chinese	Zhang et al. (2011)
			89.25	Hong Kong Han Chinese	Cheung et al. (2013)
			6.5	Indonesian	Yuliwulandari et al. (2017)
		HLA-A*31:01	25.93	European	McCormack et al. (2011)
			7.3	Korean	Kim et al. (2011)
			33.9	Japanese	Ozeki et al. (2011)
		HLA-B*15:11	18.0	Korean	Kim et al. (2011)
			9.76	Japanese	Kaniwa et al. (2010)
			54.12	Thai	Jaruthamsophon et al. (2017)
		HLA-B*15:21	40.73	Thai	Jaruthamsophon et al. (2017)
			7.53	Filipino	Capule et al. (2020)
		HLA-B*11:01	63.89	Spanish Caucasian	Ramirez et al. (2017)
	Phenytoin	HLA-B*15:02	5.1	Taiwan Han Chinese	Hung et al. (2010)
			3.50	Hong Kong Han Chinese	Cheung et al. (2013)
			18.5	Thai	Locharernkul et al. (2008)
			5.71	Malaysian	Chang et al. (2017)
		HLA-B*15:13	11.28	Malaysian	Chang et al. (2017)
		HLA-B*56:02	10.40	Thai	Tassaneeyakul et al. (2016)
		HLA-B*38:02	12.67	Thai	Manuyakorn et al. (2020)
		HLA-A*02:01/Cw*15:02	14.75	Spanish Caucasian	Ramirez et al. (2017)
		HLA-B*46:01	2.341	Thai	Sukasem et al. (2020b)
	Lamotrigine	HLA-B*15:02	5.1	Taiwan Han Chinese	Hung et al. (2010)
			4.89	Thai	Koomdee et al. (2017)
		HLA-B*15:02	3.59	Hong Kong Han Chinese	Cheung et al. (2013)
		HLA-A*31:01	11.43	Korean	Kim et al. (2017)
		HLA-A*02:07	7.83	Thai	Koomdee et al. (2017)
		HLA-B*38:01	147	Spanish Caucasian	Ramirez et al. (2017)
	Phenobarbital	HLA-A*01:01	11.66	Thai	Manuyakorn et al. (2016)
		HLA-B*13:01	4.60	Thai	Manuyakorn et al. (2016)
	Oxcarbazepine	HLA-B*15:02	80.7	Taiwan Han Chinese	Hung et al. (2010)
			27.90	Taiwan Han Chinese and Thai	Chen et al. (2017)
Antihyperuricemia	Allopurinol	HLA-B*58:01	580.3	Taiwan Han Chinese	Hung et al. (2005)
			40.83	Japanese	Kaniwa et al. (2008)
			97.8	Korean	Kang et al. (2011)
			80	89% of European Caucasian	Lonjou et al. (2008)
			348.3	Thai	Tassaneeyakul et al. (2009)
			229.7	Hong Kong Han Chinese	Chiu et al. (2012)
			203.40	Mainland Han Chinese	Cao et al. (2012)
			127.60	Mainland Han Chinese	Cheng et al. (2015a)
			579.0	Thai	Sukasem et al. (2016)
Decreasing intraocular pressure	Methazolamide	HLA-B*59:01	249.8	Korean	Kim et al. (2010)
			305	Mainland Han Chinese	Yang et al. (2016)
		HLA-Cw*01:02	22.1	Korean	Kim et al. (2010)
			12.1	Mainland Han Chinese	Yang et al. (2016)

Carbamazepine is an effective broad-spectrum anticonvulsant drug and one of the most common drugs to induce SJS/TEN. Carbamazepine–induced SJS/TEN was first shown to be strongly associated with HLA-B*15:02 in Taiwan Han Chinese (Chung et al., 2004). Later, this strong association was confirmed in other ethnicities including Thai, Indian, mainland Han Chinese, and Malaysian, but not in European, Korean, Japanese, and Filipino populations. Individuals at highest risk of Han Chinese descent, followed by those in Vietnam, Cambodia, the Reunion Islands, Thailand, India (specifically Hindus), and Malaysia, are recommended to be screened for the presence of HLA-B*15:02 allele prior to starting carbamazepine by Clinical Pharmacogenetics Implementation Consortium (CPIC) guidelines (Leckband et al., 2013). HLA-A*31:01 and HLA-B*11:01 were reported to be associated with carbamazepine–induced SJS/TEN in Caucasians (McCormack et al., 2011; Ramirez et al., 2017). HLA-A*31:01 and HLA-B*15:11 were reported to be associated with carbamazepine–induced SJS/TEN in Korean and Japanese (Kaniwa et al., 2010; Kim et al., 2011; Ozeki et al., 2011). HLA-B*15:21 was associated with carbamazepine–induced SJS/TEN in Filipino (Capule et al., 2020). Another example is the antiseizure drug phenytoin induced SJS/TEN. In Malaysia, it was not only found to be associated with HLA-B*15:02 but also with HLA-B*15:13 (Chang et al., 2017). In Thai population, HLA-B*15:02, HLA-B*56:02, HLA-B*38:02, and HLA-B*46:01 were observed to be associated with phenytoin-induced SJS/TEN (Locharernkul et al., 2008; Tassaneeyakul et al., 2016; Sukasem et al., 2020b; Manuyakorn et al., 2020). When prescribing phenytoin to patients, CPIC guideline recommended considering the HLA-B*15:02 genotype first, then other variants such as CYP2C9 and HLA-A*31:01 (Karnes et al., 2020).

Phenytoin, phenobarbital, lamotrigine, carbamazepine, and oxcarbazepine are commonly used antiepileptic aromatic amines and they have similar chemical structures. HLA-B*15:02 is the common risk allele for aromatic antiepileptic. The patients carrying HLA-B*15:02 in phenytoin, lamotrigine, or oxcarbazepine SJS/TEN sufferers, took proportion of 30.8% (OR = 5.1), 33% (OR = 5.1), 100% (OR = 80.7) respectively, in a case-control study (Hung et al., 2010). This indicates that the aromatic antiepileptic-drugs share common risk allele, and HLA-B*15:02 is the most significant. Take aromatic antiepileptics as a whole, HLA-A*24:02 was identified as another independent risk factor for SJS/TEN in Southern Han Chinese patients, and this strong association was not only in aromatic antiepileptics but also in each individual drug including carbamazepine, lamotrigine, and phenytoin (Shi et al., 2017). The study also revealed a multiplicative interaction between HLA-B*15:02 and HLA-A*24:02. Positivity for HLA-A*24:02 and/or HLA-B*15:02 showed a sensitivity of 72.5% and a specificity of 69.0%.

Allopurinol, a drug widely used to treat hyperuricemia and its complications, is commonly reported to induce SJS/TEN. A research team from Taiwan first found the risk of allopurinol-induced severe skin reactions is 580.3 times higher in HLA-B*58:01 carriers than noncarriers in Han Chinese patients (Hung et al., 2005). They recruited 51 individuals with allopurinol-induced severe allergic skin reactions, all having the HLA-B*58:01 genotype (100%), while in the control group containing 135 individuals tolerant with allopurinol (using allopurinol at least six months without adverse responses) and 93 healthy subjects, the probability of carries with the HLA-B*58:01 was, respectively, 15% (20/135) and 20% (19/93). Afterward, a number of studies showed HLA-B*58:01 is a high-risk factor of allopurinol-induced skin reactions in Mainland Han Chinese (Cao et al., 2012; Chiu et al., 2012; Cheng et al., 2015a), Thai (Tassaneeyakul et al., 2009; Sukasem et al., 2016), Korean (Kang et al., 2011), European (Lonjou et al., 2008), and Japanese (Dainichi et al., 2007; Kaniwa et al., 2008), with 229.7, 203.40, 127.60 (Han Chinese), 348.3 (Thai), 97.8 (Korean), 80 (European), and 40.83 (Japanese) fold risk than noncarriers. It seems there is a universal risk HLA genotype with allopurinol-SJS/TEN, but to varying degrees, with a higher association in Han Chinese and Thai, and a lesser but significant association in other ethnicities like Japanese, Korean, and European. A study in Korean revealed that HLA-B75, DR13 homozygosity, and DR14 increased the risk of allopurinol-induced SCARs when combined with HLA-B*58:01, especially in those with impaired kidney function (Shim et al., 2019). To reduce the risk of allopurinol hypersensitivity syndrome, CPIC guidelines recommend testing for the HLA-B*58:01 allele in populations of Korean descent with stage 3 or worse chronic kidney disease and those of Han Chinese or Thai descent prior to initiation of the drug (Saito et al., 2016).

Except for those commonly reported drugs, some other drugs are described to be associated with HLA. Strontium ranelate, a medicine to treat osteoporosis, was associated with HLA-A*33:03 and HLA-B*58:01 in Han Chinese in Singapore (Lee et al., 2016). In the context of methazolamide (a carbonic anhydrase inhibitor to lower intraocular pressure)-induced SJS/TEN, HLA-B*59:01 was a strong risk in Koreans (Kim et al., 2010) and Han Chinese (Yang et al., 2016). Ueta et al. reported that cold medicine (including NSAIDs and multi-ingredient cold medications)-induced SJS/TEN with SOC was strongly associated with HLA-A*02:06 (OR = 5.6) and HLA-B*44:03 (OR = 1.99) in Japanese patients (Ueta et al., 2014a). In addition, the authors found HLA-B*44:03 was significantly associated with cold medicine-SJS/TEN with SOC in the Indian, Brazilian, and Thai, but not the Korean population, and that HLA-A*02:06 might be weakly associated in the Korean but not the Indian and Brazilian population (Ueta et al., 2014b; Jongkhajornpong et al., 2018; Jun et al., 2019). Except for these two alleles, a meta-analysis revealed HLA-A*33:03 (OR = 2.28) and HLA-C*05:01 (OR = 2.55) were also associated with cold medicine-induced SJS/TEN with SOC (Tangamornsuksan et al., 2020). Furthermore, trimethoprim/sulfamethoxazole- (TMP/SMX-) induced SJS/TEN was significantly associated with HLA-B*15:02 and HLA-C*08:01 in Thai patients (Kongpan et al., 2015; Sukasem et al., 2020a). As more and more HLA markers have been found to be associated with drug-induced SJS/TEN, prospective screening for HLA genotypes to avoid drug hypersensitivities is highly recommended (Chen et al., 2011; Saokaew et al., 2014; Cheng et al., 2015b; Dong et al., 2015; Ko et al., 2015; Sukasem et al., 2018b).

There are four major hypotheses or models to predict antigen presenting in drug-induced SJS/TEN (Pichler et al., 2011; Duong et al., 2017). **i) Hapten/prohapten model:** the “hapten and prohapten model” thinks the drug or drug metabolites bind covalently to a peptide carrier and then are presented by HLA proteins to TCR in antigen presenting cells (APC). One example of hapten model is penicillin metabolites binding to serum albumin, generating chemically modified peptide and triggering the immune reaction (Padovan et al., 1996). **ii) Noncovalent, direct binding of the drug to a peptide (pharmacological interaction (p-i) model):** the “p-i model” thinks the drug or drug metabolites directly or noncovalently bind to HLA protein and are presented to TCR without a peptide carrier-loading. One example is carbamazepine-induced SJS/TEN. Carbamazepine binds to HLA-B*15:02 directly without any peptide assistance, as evidenced by the fact that fixation of APC could still elicit an immune reaction (Wei et al., 2012). In “p-i model”, APC or intracellular antigen-processing pathway is not necessary to elicit the immune response. **iii) Altered peptide repertoire model:** X-ray crystallography illustrates that the drug binds directly to the HLA and alters its specificity, resulting in presentation of novel ligands and leading to cytotoxic T-cell activation. One example is the abacavir-induced hypersensitivity. Abacavir alters the repertoire of self-peptides and changes the binding cleft of the HLA-B*57:01 protein, where it acts as the foreign antigen resulting in generation of a polyclonal T-cell response and induction of hypersensitivity reactions (Illing et al., 2012; Ostrov et al., 2012). **iv) Altered TCR repertoire model:** in contrast to the third model, some drugs can bind directly to TCR without peptide-HLA complex on APC. One example is sulfamethoxazole that causes change in TCR conformation, free from HLA or peptide interaction (Watkins and Pichler, 2013). Taken as a whole, no matter which model is used to predict antigen presenting, it is still not well understood how HLA emerge into drug-specific immune-response so far.

## T-Cell-Mediated Apoptosis Signaling Pathways in SJS/TEN

**FIGURE 2:**
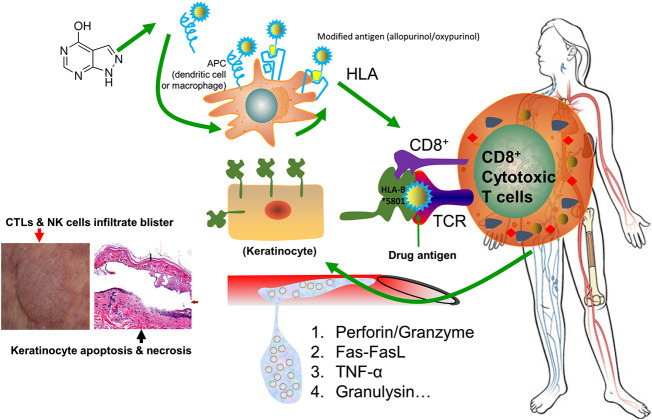
Schematic representation of immunopathogenic mechanism underlying drug-induced SJS/TEN. The drug allopurinol is listed as the demonstrating drug. Allopurinol and/or its active metabolite oxypurinol is presented by antigen presenting cells (APC) and is interacting with HLA-B*58:01 protein. They are capable of generating a sufficient strong signal to TCR for cell activation. Upon activation, the stimulated CD8^+^ cytotoxic T cells will have a cascade release of cytokines or chemokines, including perforin/granzyme, Fas-FasL, TNF-α, and granulysin, which will kill keratinocytes and mucosal cells causing skin sloughing and necrosis. Meanwhile, CTLs and NK cells will infiltrate into the skin to form blisters. The left bottom image illustrates keratinocyte necrosis (black arrow) and development of large bullae (red arrow), adapted from (Gupta et al., 2019). Abbreviations: APC: antigen presenting cells. APCs include dendritic cells, macrophages, Langerhans cells, and B cells. TCR: T-cell receptor. CTLs: cytotoxic T lymphocytes. NK cells: natural killer cells.

Drug-induced SJS/TEN is generally believed to be an HLA restricted T lymphocyte cytotoxicity (Chang et al., 2020). The drug activates the natural killer (NK) cells and cytotoxic T lymphocytes (CTLs), and their effect is largely amplified by cascade release of death mediators. Perforin and granzyme, Fas–Fas ligand, TNF-α, and granulysin are the most frequently reported mediators involved in SJS/TEN. As T lymphocytes infiltrate into the lesions, it shows cell toxicity to autologous target cells and leads to massive death of keratinocytes and mucosal cells. A schematic representation of immunopathogenic mechanism underlying drug-induced SJS/TEN is shown in Figure 2.

### Perforin-Granzyme B Create Channels in the Cell Membrane and Activate Cell Death

Perforin and granular enzyme are proteins that play a part in cytotoxicity. These macromolecules are usually expressed in CTLs and NK cells and are subject to regulation of several cytokines such as IL-2 and IL-12. When the activated CTLs and NK cells bind to target cells, effector cells release dense cytoplasmic granules to the binding sites between target cell and effector cell through granule exocytosis. The most important macromolecules released in the degranulation process are perforin and granzyme B. A high expression of granzyme B in CD8^+^ T cells was detected in blisters (or skin) samples of SJS/TEN (Chung et al., 2008). Granzyme B is released along with perforin which inserts into a target cell's plasma membrane forming a pore. Perforin is a pore forming cytolytic protein secreted by CTLs and NK cells. Upon degranulation, perforin changes from monomer molecular to the conformation that exposes the dichotomy region of hydrophilic molecules, in a Ca^2+^-dependent manner. Several monomers are polymerized to form transmembrane channels, which are 5–20 nm in diameter. The hydrophilic monomer amino acid residues are located at the inside of these channels, while the hydrophobic residues are sited toward the phospholipid bilayer (Liu et al., 1996). And thus, perforin is capable of inserting into the lipid bilayer of the target cells. After the channel loses ion selectivity, the water, ions, and other small molecules can move freely across the tunnel into the target cells. Finally, the osmotic shock will cause cell swelling which will end up to burst or apoptosis.

### Fas–Fas Ligand Interaction Destroys Keratinocytes

Fas and Fas ligand are well-deliberated apoptosis-related membrane surface molecules. The Factor associated suicide (Fas) and Fas ligand (FasL) pair function as a guardian against autoimmunity and tumor genesis. In 1989, Yonehara et al. (Yonehara et al., 1989) found a monoclonal antibody which recognizes the unknown molecule in the surface of myeloid cells, T lymphocytes, and fibroblasts, and could induce apoptosis in many human cell lines. This new antigen was named as Fas. In 1993, Suda et al. (Suda et al., 1993) successfully cloned FasL from the CTLs hybridoma-derived cell line PC60-d10S cells, which are capable of binding to Fas and subsequently mediate cell apoptosis. In 1998, Viard and colleges (Viard et al., 1998) reported Fas-FasL can mediate apoptosis of keratinocytes. In SJS/TEN, FasL was upregulated before clinical development of skin detachment and mucosal erosions, and the concentration was much lower than that of granulysin (Murata et al., 2008). In general, FasL in CTLs will bind to Fas in target cells, triggering apoptosis through Fas-associated protein with death domain (FADD) adaptor-mediated recruitment of a series of downstream factors, including Caspase-8, resulting eventually in cell apoptosis.

Before Fas/FasL was found, it was considered that the killing effect of CTLs was largely accomplished by perforin and granzyme. Now we know that perforin/granzyme B is working independent of Fas-FasL to cause cell apoptosis. Perforin can punch pores into the target cell membrane in the presence of Ca^2+^. However, in the absence of Ca^2+^, activated CTLs can still kill target cells via Fas/FasL. Upon CTLs recognition of target cells, Fas is highly expressed on cell surface of target cells (like keratinocytes) and the ligation of Fas and FasL will trigger the apoptotic program to release a caspase cascade; subsequently the target cells are subject to programmed cell death. In addition, the FasL interaction could promote the inflammation via NF-κB activation (Ahn et al., 2001; Xiao et al., 2002).

Additionally, diverging results remain as Chen and colleagues found the pathological feature of chronic conjunctivitis in SJS patients was consistent with chronic inflammation, but the expression of Fas-FasL in conjunctiva was as low as normal conjunctiva (Chen et al., 2014). It seems Fas-FasL is unlikely to mediate conjunctival cell death in SJS/TEN. Whether the elevation of serum Fas-FasL is responsible for the death of conjunctival and mucous cells needs further investigation.

### TNF-α-Related Apoptosis

TNF-α, a substance made by monocytes and macrophages in the epidermis, has been identified as a mediator of keratinocyte death. TNF-α can interact with a surface death receptor TNF receptor-1, which will induce the recruitment of adaptor proteins such as FADD to trigger the extrinsic apoptotic pathway, leading to cell death. Plenty of studies support that TNF-α is strongly expressed in SJS/TEN lesions and is involved in the epidermal necrosis (Caproni et al., 2006; Neuman and Nicar, 2007; Wang et al., 2018b). Excitingly, TNF-α antagonists are translated into clinical therapeutics for SJS/TEN in recent years. Tons of evidence supports TNF antagonists halt SJS/TEN (Paradisi et al., 2014; Gavigan et al., 2018; Shear et al., 2018; Trujillo-Trujillo et al., 2018; Chafranska et al., 2019; Coulombe et al., 2019; Pham et al., 2019; Zhang et al., 2020). A randomized controlled trial compared etanercept (a TNF inhibitor) with systemic corticosteroid treatment in adult SJS/TEN patients and showed a decreased SCORTEN-predicted mortality rate, reduced skin healing time, and less gastrointestinal hemorrhage in the etanercept group. In addition, TNF-α and granulysin secretion in blister fluids and plasma were decreased by 45.7–62.5% and the regulatory T cells (Tregs) population was increased by two fold (Wang et al., 2018a). However, the risk of serious infection is increased when using potent immunosuppressant, and thus the risk of TNF inhibitors should be weighted when administrated (Woolridge et al., 2018).

### Granulysin, the Cytolytic Granules, Mediates the Keratinocyte Apoptosis

Analysis of blister fluids from carbamazepine, phenytoin, and amoxicillin-induced SJS/TEN in Han Chinese has pointed out that a high expression of granulysin secreted by CTLs and NK cells is the key molecule responsible for the disseminated keratinocyte death in SJS/TEN (Chung et al., 2008). There was a high amount of granulysin in the blister fluid and its concentration was 2–4 times more than other cytotoxic proteins. The expression of granulysin was high in the plasma even before the onset of blister and slough. Granulysin was found to have a strong role in cell membrane perforation triggering cell apoptosis, and dissolving a wide variety of bacteria and killing tumor cells. When injecting granulysin into the mouse skin, the mice developed a sign similar to SJS/TEN. For this reason, it is believed that granulysin plays an important role in regulating epidermal cell death in SJS/TEN (Saito et al., 2013).

As granulysin is the predominant cytotoxic protein in SJS/TEN, therapies targeting granulysin may be promising for SJS/TEN. A recent study showed that the c.11G > A heterozygous mutation in granulysis encoding gene contributed to a mixed drug-induced SJS/TEN. This mutation creates a premature termination codon (p.Trp4Ter) and an abnormal subcellular mislocalization of granulysin (Fonseca et al., 2019). A group in Taiwan developed a novel siRNA chimera targeting granulysin and CTLs that showed great effect on inhibiting granulysin in activated T cells (Wang et al., 2013). Though not tested in patients, this method may lay a cornerstone for future research on granulysin.

### Other Cytokines or Molecules

Except for the cytokines mentioned above, other cytokines including IFN-γ and IL-15 are involved in the development of SJS/TEN and might be used as biomarkers for SJS/TEN. A report showed a significant higher serum levels of Th1 cytokine IFN-γ and chemokines CXCL9 and CXCL10 in SJS/TEN patients than tolerant controls (Wang et al., 2016). IL-15 is a proinflammatory cytokine that plays an essential role in the activation and proliferation of T cells and NK cells. Su et al. found IL-15 was significantly associated with SJS/TEN fatalities (Su et al., 2017). Notably, long-standing evidence has suggested that IL-15 could be a diagnostic and prognostic marker, and a therapeutic target for SJS/TEN (Stern and Divito, 2017), though not tested in clinical settings. Serum level of IL-33, which might be released from epidermal keratinocytes, was elevated in patients with TEN in the early stage and gradually decreased during their clinical course. Therefore, IL-33 might be a good marker for the early stage of TEN (Adachi et al., 2019).

Besides these, Hama et al. used the mass spectrometry to analyze the supernatant in PBMCs from SJS/TEN patients after reexposure to causative drugs and found galectin-7 was strongly upregulated (Hama et al., 2019). Galectin-7 is a protein specifically expressed in keratinocytes and is moderately repressed by retinoic acid (Magnaldo et al., 1995). High-mobility group box 1 protein (HMGB1) is a damage-associated molecular-pattern protein. Serum level of HMGB1 was found significantly higher in SJS/TEN compared with healthy controls in Japanese (Fujita et al., 2014) and Taiwanese patients (Carr et al., 2019), but its expression level varied on a case-to-case basis (Adachi et al., 2019).

The kinetics of cytokines varies tremendously during the disease course. For instance, the concentrations of soluble FasL and granulysin decrease rapidly in the disease course (Murata et al., 2008; Abe et al., 2009), while IFN-γ and TNF-α quantities are relatively stable (Yang et al., 2017; Lerch et al., 2018). The variable levels of these cytokines in SJS/TEN depend largely on the relative disease course (Yang et al., 2017), and some of them can decrease immediately after initiation of immunosuppressive therapy albeit no improvement in the skin symptoms. Therefore, finding a good cytokine marker that correlates with disease severity and mortality but may not be interfered by treatment is urgently needed for the diagnosis and prognosis of SJS/TEN.

### Regulatory T Cells

Tregs cells are essential in regulating immune homeostasis and inflammation (Chen and Oppenheim, 2014; Zhou et al., 2014). Treg inhibits the function of T effector cells (Teff) at the site of inflammation, thus limiting severe immunopathology (Takahashi et al., 2009). A previous study showed Tregs were almost lacking in the SJS skin lesion (Amakata and Teraki, 2019), and its expression could prevent life-threatening skin damage such as TEN (Azukizawa et al., 2005). A study in Japan suggested that the infection affected the activity of Treg and caused more severe symptoms of SJS/TEN and early onset time (Okamoto-Uchida et al., 2017). A study explored Tregs in allopurinol-induced SJS/TEN and found allopurinol suppressed the chemokines involved in Treg migration and compromised the migratory chemokine receptors in human keratinocyte cell lines, highlighting the sparse distribution of Tregs in the skin of SJS/TEN patients (Osabe et al., 2018).

## Discussion and Future Perspective

As the most severe adverse drug reactions with high mortality, SJS/TEN is initiated jointly by environmental and genetic factors, and the interaction between these two. However, in about half cases of SJS/TEN no causative drug is identified (Auquier-Dunant et al., 2002) and in 15% of SJS/TEN patients they either had taken no drug or drug responsibility is deemed unlikely (Sassolas et al., 2010). Virus, bacterial, or mycoplasma infections are suggested to be a trigger for SJS/TEN. Some reported autoimmune diseases such as lupus erythematous and dermatomyositis without any drug causality or infectious aetiology can be a cause of SJS/TEN (Ziemer et al., 2012; Dumas et al., 2018). Finding a common allogeneic etiology that can elicit SJS/TEN is imperative to further elucidate the pathophysiology of SJS/TEN. Even with a variety of genetic markers being used for prophylaxis, new diagnosis of SJS/TEN is made every year. Various *in vivo*, *in vitro*, and *ex vivo* assessment tools have been exploited to aid in finding the causative drugs and predict the prognosis. For instance, drug-challenged *ex vivo* lymphocyte transformation test such as IFN-γ ELISpot has been adopted to identify possible culprit drugs in SJS/TEN (Klaewsongkram et al., 2016; Trubiano et al., 2018).

Notwithstanding the fact that numerous researches have been input to autoimmune reactions and HLA serotypes, it is worth mentioning that the correlation between SJS/TEN and HLA does not mean immunological reactions are the sole effector mechanism of epidermal necrosis. Other genetic or metabolic factors outside HLA need to be delineated. Some studies have found the possible roles of epigenetics, including microRNAs, ncRNAs (noncoding RNA), and DNA methylation, in drug-SJS/TEN (Ichihara et al., 2014; Sato et al., 2015; Sun et al., 2017; Cheng et al., 2018). Mawson and colleagues proposed SJS and TEN share a common pathogenesis that it is endogenous retinoids (vitamin A and its congeners) that obstruct normal liver function and spill to the circulation that cause SJS/TEN. Blocking circulating retinoids could mitigate the symptoms of SJS/TEN (Mawson et al., 2015). A study showed Wnt signaling was downregulated in drug-induced SJS/TEN, and enhancement of Wnt signaling attenuated *ex vivo* activation of drug-specific T cells from SJS/TEN patients (Chen et al., 2020). Plus, using metabonomics to study the metabolites might be helpful to identify the common etiology to cause SJS/TEN. Mobilization of Tregs and expansion of tolerogenic myeloid precursors might promote reepithelialization on SJS/TEN (Wolkenstein and Wilson, 2016), and this could be another research direction.

Although gratifying achievements have been made, pivotal questions on pathogenesis of SJS/TEN underlying gene polymorphisms and T cell cytotoxicity remain. For instance, why some of the patients carrying the risky genes tolerate the drug and do not develop SJS/TEN? What makes the skin and mucous membrane in the mouth, throat, eyes, genitals, and anus so special to be targeted in SJS/TEN? Do they relate to skin expression of drug transporters? What is the link between various culprit drugs? Do risky HLA-B alleles work by the same mechanism? And is there a common risk factor or metabolite among these drug hypersensitivities? In addition to well-elucidated HLA and CYP variants, more studies are warranted to reveal novel risk factors and mechanisms to promote diagnosis, treatment, and prognosis of SJS/TEN. Pharmacogenetics will be sure to play a big role in the prevention and individualized treatment of severe adverse drug reactions.
